# MR‐Guidance of Gene Therapy for Brain Diseases: Moving From Palliative Treatment to Cures

**DOI:** 10.1002/jmri.29804

**Published:** 2025-04-21

**Authors:** Dalton H. Bermudez, Thomas Lilieholm, Walter F. Block

**Affiliations:** ^1^ Department of Medical Physics UW Madison Madison Wisconsin USA; ^2^ Department of Biomedical Engineering UW Madison Madison Wisconsin USA; ^3^ Department of Radiology UW Madison Madison Wisconsin USA

## Abstract

Regulatory bodies in the U.S. and Europe recently approved a gene therapy for aromatic L‐amino acid decarboxylase (AADC) deficiency, a rare neurologic disorder where a genetic mutation prevents dopamine production in the brain. Affected children fail to develop normal motor and cognitive functions. MRI‐guided intraparenchymal delivery of AADC gene therapy to localized gray matter regions—specifically the substantia nigra and ventral tegmental area—has enabled the brain to produce dopamine, resulting in dramatic improvements in physical and cognitive outcomes. The need to target only a small brain region simplifies the surgical approach. However, gene therapy for broader neurodegenerative conditions has progressed more slowly than expected, despite significant global investment. Clinical efficacy depends heavily on the accurate delivery of gene therapeutics via direct brain infusion, cerebrospinal fluid (CSF) administration, or both. Inadequate image guidance during clinical trials makes it difficult to distinguish between true drug inefficacy and delivery failure. We highlight how increasing use of MRI for pre‐surgical simulation and real‐time therapy monitoring is accelerating gene therapy development for neurological diseases. This manuscript explores MRI's role in guiding intraparenchymal gene delivery, particularly using Convection Enhanced Delivery (CED). MRI contributes across the treatment timeline—from pre‐surgical planning and infusion guidance to validating therapeutic coverage. We describe how MRI supports controlled therapeutic distribution for localized treatments and its potential to enable broader distributions needed for correcting widespread genetic anomalies. We also detail how structural and anatomical MRI sequences (T1, T2, Time of Flight, and Diffusion Tensor Imaging (DTI)) can help model likely infusion distributions. Finally, we provide an outlook on how advanced DTI‐based algorithms and poroelastic theory could further improve modeling of infusion dynamics. Current MRI‐based technologies can be integrated and enhanced to improve CED effectiveness, especially in very young pediatric patients.

**Evidence Level:**

1.

**Technical Efficacy:**

Stage 4.

## 1 Introduction

Advances in gene therapy for neurodegenerative diseases over the last 30 years have spurred a multibillion‐dollar pharmaceutical segment, international regulatory cooperation, and approximately 50 clinical trials in the United States alone [1]. Adeno‐associated viruses (AAVs) [2, 3] are often employed to deliver genetic material to targeted central nervous system (CNS) regions in hopes of treating debilitating diseases such as dementia, Parkinson's Disease (PD), and numerous other rare, often fatal, disorders. A recent powerful example of successful gene therapy has seen children facing a catatonic life due to a rare condition that does not allow them to produce dopamine, known as aromatic L‐amino acid decarboxylase deficiency (AADC), suddenly catching up on motor and cognitive developmental milestones after treatment. Although newly approved treatments in sickle cell therapy have [4] garnered wider attention, the number of approvals is lagging behind that which has been predicted over the past few years by the FDA and regenerative medicine community [5] Slow adoption and development of image‐guided therapy is a partial cause of this slow rollout.

To avoid the blood–brain barrier (BBB) in diseases affecting the CNS, trials often first use an intrathecal, intracisternal or intraventricular delivery approach [1] as this involves less risk, regulation, and clinical cost than a direct brain approach. Intrathecal therapy works well particularly in the spinal cord of very young Spinal Muscular Atrophy (SMA) patients as the therapy can penetrate the small diameter of the infant spinal cord [6]. Bi‐weekly intraventricular treatments of enzymatic replacement [7] (Brineura, BioMarin) have been shown to slow metabolic diseases like Batten Disease (NCL) but the penetration of gene therapies into the brain itself via these outside‐in pathways is too limited to stop the fatal progression of these neurodegenerative diseases [5]. Although MRI has shown some value in validating the local distribution of cerebral spinal fluid (CSF)‐based delivery, the relatively sparse infusate distribution at modest distances from the initial point of therapeutic delivery makes monitoring with MRI of limited value with intrathecal/intracisternal/intraventricular delivery.

**FIGURE 1:**
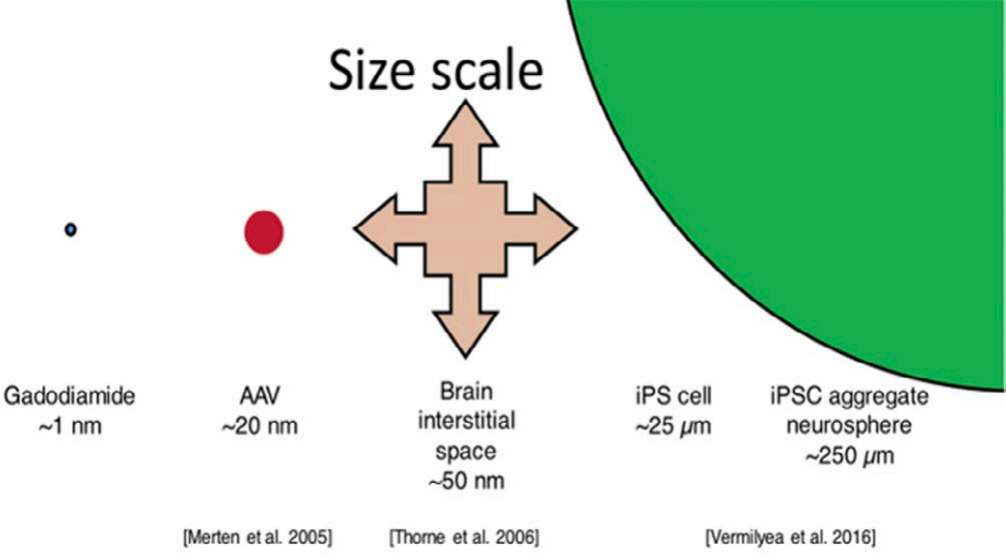
The relative sizes of administered therapeutic adeno‐associated viral vectors (AAVs) (~20 nm) relative to the width of interstitial spaces in the brain (~50 nm) enables therapeutic agents to diffuse through interstitial spaces after CED injection has circumveted the blood brain barrier and circumvent the blood brain barrier Induced Pluripotent Stem Cells (iPSC) (~250 μm)cannot diffuse through interstitial spaces [9, 10, 11].

Instead, direct intraparenchymal delivery is increasingly used to administer gene therapies in numerous lysosomal storage diseases (Batten, Tay‐Sachs, Gaucher, Niemann‐Pick, Sanfilippo), leukodystrophies, and ataxias [8]. In these treatments, minimally invasive catheters are placed in the brain through small burr holes drilled through the skull. As shown in Figure 1, the nominally small size of AAV vectors (20 nm) permits passage through interstitial pathways on the order of 50–100 nm [9]. Their size supports expansion of the infusion if done slowly under pressure. Infusion rates are only on the order of microliters/min [12]. Neurosurgeons first developed CED [13, 14] to deliver chemotherapeutic agents in tissue adjacent to resected malignant brain tumors and recurrent brain tumors. In these procedures, MRI [15] is used for the pre‐surgical planning of catheter placement [16] and post‐surgical validation of the therapeutics' distribution, but it was rarely used to monitor during infusions, even though failure to deliver therapeutics to the intended regions has been used to explain the failure of some trials [17].

Past trials have often been initiated with MRI playing little role in the guidance [18] and monitoring of therapeutic distribution. The first failed trials of gene therapy to deliver neurotherapeutic agents for Parkinson's Disease in the striatum demonstrated the need for monitoring the distribution of infusate in real time [4]. In one post‐trial autopsy, only 20% of the putamen, the intended target, was found to have been treated. Repeating expensive PD trials becomes necessary if the trial cannot determine if the therapeutic itself failed or if it simply was not delivered to the intended region. In rare metabolic diseases, sampling CSF can show if a therapy had some effect in creating a necessary new protein. But without knowing which region of internal anatomy has received the therapeutic agent, clinicians and parents can only wait anxiously to see what clinical effect will result. Due to this, MRI monitoring [19] is increasingly necessary in clinical trials to warrant their expense and deliver an adequate risk/benefit ratio [20, 21].

In some devastating rare monogenic neurodegenerative diseases, missing genes simply need to be added and not edited into the genome. For example, in the most prevalent form of Batten Disease (CLN2), a gene needed to build an enzyme for metabolic waste clearance, tripeptidyl peptidase 1 (TPP1) is missing. Several groups have built viral vectors or nanoparticles that produce the missing enzyme, but clinical trials have failed to deliver these solutions to broad enough regions of the brain [22]. As most AAV‐based gene therapy molecules are compact enough to flow wherever the pressurized buffer carrying them flows, the remaining gene therapy delivery issues can largely be addressed as a biophysics problem of fluid movement, rather than a pharmacokinetic problem. Simply put, the central drug delivery issue vexing therapies for neurodegenerative diseases affecting wide expanses of the brain revolves around forcing the therapeutic's buffer to flow throughout heterogeneous brain tissue before it is shunted out via sulci and perivascular (PVS) spaces. As MRI is well‐suited to querying and visualizing changes in water movement and fine brain anatomy, the modality has made key contributions to date and is poised to play a significant role in solving remaining gene therapy distribution shortfalls.

We first highlight the MR methodologies that are guiding catheters and monitoring therapies where the therapeutic target has been localized to a homogenous gray matter region. Therapeutic strategies where the treatment region can remain localized within gray matter are simplified as the tissue's interstitial flow parameters are largely isotropic. Planning infusions that cover wide expanses of gray and white matter is more complicated, as the flow parameters dictating the expansion of the infusion in white matter are highly anisotropic. We next outline methods to predict the final distribution using principles of poroelastic fluid transport through a porous medium [23]. Although the maps of hydraulic parameters needed to forward model the infusion expansion cannot be directly measured non‐invasively, we show how clever use of MRI protocols can provide surrogates of the necessary hydraulic parameters. We then describe how advanced structural and diffusion tensor imaging (DTI) methodologies could provide more direct characterization of the needed hydraulic parameter maps. We conclude with recommendations on how further research within the MR brain mapping community could enable next‐generation modeling of gene therapy trials that must treat much larger brain volumes before the disease degrades the entire brain and/or cerebellum. Current review articles on gene therapy studies focus on the molecular biology of new gene therapeutic agents, neurosurgical methods, and clinical outcomes. We instead first focus on how MRI is used to effectively deliver gene therapy in trials designed to cover localized areas of gray matter. We then discuss how further development in MRI and viral vector delivery is needed to overcome hurdles in diseases that require whole brain coverage.

## 2 Viral Vectors for MRI Guidance of Intraparenchymal Gene Therapy

AAV vectors are the predominant gene delivery platforms used in CNS gene therapy, with 87% of clinical trials utilizing this approach due to their small size, lack of replication of the viral vector and pathogenicity, potent neuronal tropism, and longevity of gene expression in nonreplicating cells. AAV9 has received a lot of attention in pre‐clinical experiments for its ability to cross the BBB [24] and permeate the spinal cord in SMA studies, with some Investigational New Drug (IND)‐initiating work in intrathecal administration to warrant trials in human neurodegenerative diseases [25]. The practical issue of creating doses large enough for intravenous administration becomes apparent, however, when models reach the size of a rhesus macaque [24]. AAV9, therefore, will likely be useful for inexpensive pre‐clinical research of new agents, but will not obviate the need for intraparenchymal delivery.

The tight correspondence between MR imaging of the infusion buffer and the resultant gene expression has been demonstrated in multiple studies [26]. Rather than being a pharmacokinetic problem, controlling the infusion zone such that it expands throughout the brain's porous tissue relies more on the biophysics of transport through a porous medium.

Although improved viral vectors are creating larger volumetric distributions with CED, the increases in transduction efficiency are moderate compared to the increases in volumetric coverage needed for diseases affecting the entire brain and/or cerebellum. Thus, leaders in the field have called for further investment in vector development and image‐guidance technology to move from palliative approaches that merely slow progression to actual cures [19]. Regardless of vector development, MRI will continue to be central in monitoring and assessing gene therapy distribution.

## 3 Overview of MRI'S Role in Planning and Executing Intraparenchymal Gene Therapy

The catheters used in gene therapy delivery are generally non‐metallic. The signal voids of these catheters are normally used to precisely determine the orientation and location of the devices. This visualization capability builds off prior contributions to accurately track biopsy needles [27, 28]. These contributions mitigated displacement artifacts [29, 30] that varied with MR pulse sequences and the relative offset between needle orientation and the B0 field direction [31].

**FIGURE 2:**
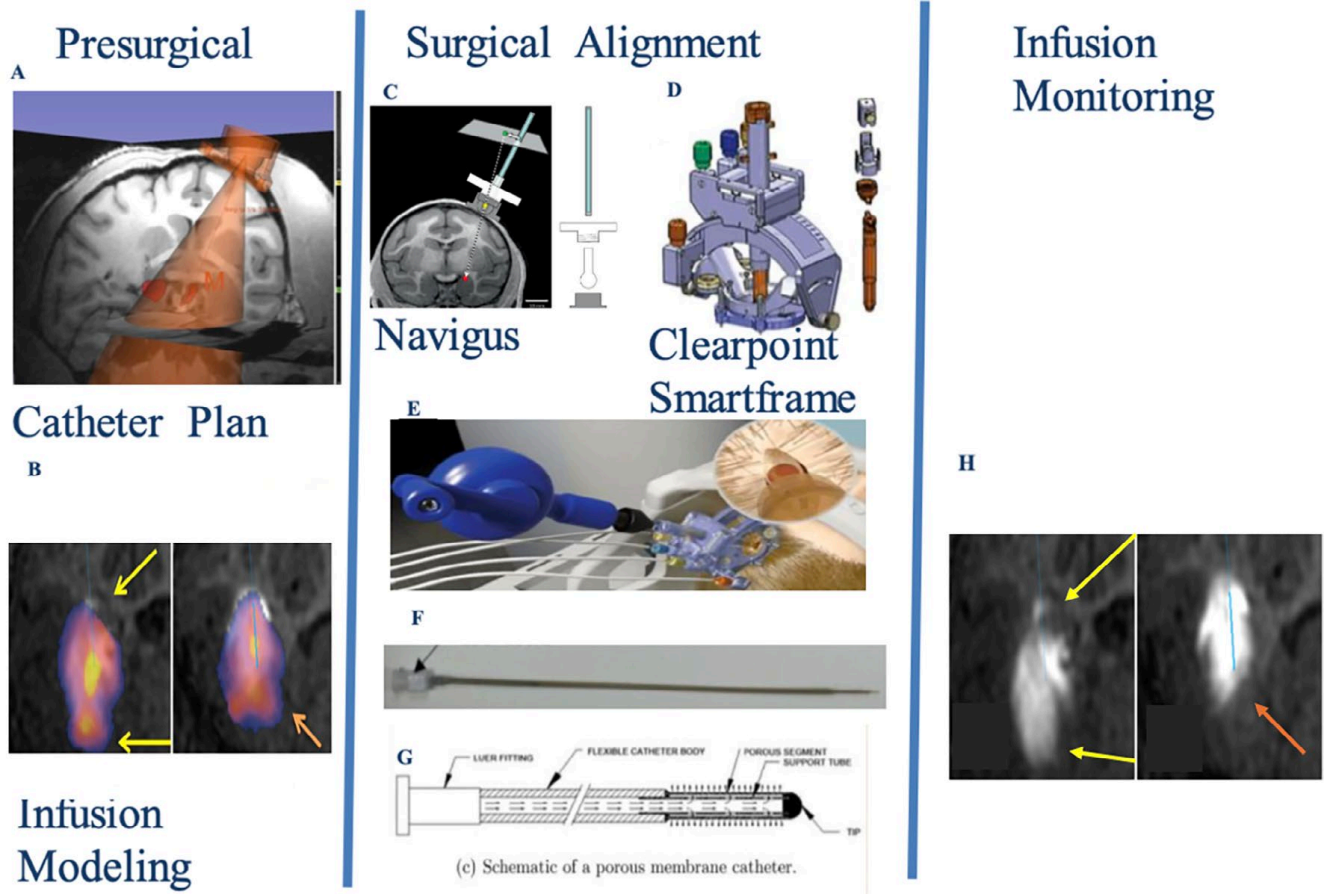
Presurgical workup involves (A) planning multiple safe catheter trajectories and (B) forward modeling the infusion distribution expected to result from each catheter location. (C) Guiding catheter placement is performed in the stereotactic operating room using brain ports like the medtronic navigus on the medtronic's stealthstation surgical navigation system or brainlab navigation system. When added precision is needed, catheters are placed in the MRI suite with the (D‐E) ClearPoint SmartFrame. Single endport catheters are normally used including the (F) SmartFlow (ClearPoint Neuro, Irvine, CA). (G) Porous membrane catheters (Occam, Louisville, KY) have shown promise in creating larger distributions in preclinical trials. (H) MRI‐based gene therapy largely uses T1‐weighted imaging that is sensitized to co‐infused Gadolinium (Gd) contrast agents [23, 32, 33, 34].

The applications of MRI in surgical planning, catheter guidance, and monitoring CED infusions are shown in Figure 2. Catheter trajectory planning (Figure 2A) is possible on commercial platforms including Stealth (Medtronic), BrainLab, and ClearPoint Neuro (Irvine, CA) workstation. The catheters used in gene therapy are made from non‐metallic materials such as plastic or silicone. As a result, they appear as signal voids in imaging without causing displacement artifacts, allowing for simpler monitoring during treatment [31, 35, 36, 37]. The prospective stereotactic technique was first described by Truwit in 2001 [30] and expanded upon by Martin and Truwit et al. in 2008 in JMRI [37]. The concept worked by imaging a plane perpendicular to the desired trajectory. The trajectory was aligned when the MRI‐visible cannula surrounding the catheter pathway intersected the perpendicular plane at the desired location. Prospective stereotaxy remained the predominant method for MRI‐guided targeting and localization in research studies until the advent of the ClearPoint SmartFrame [37].

Using these tools, the neurosurgeon considers the issues of each surgical approach, such as screwing the brain trajectory guide to the curved skull and avoiding conflicts with the vasculature, the midline, and the ventricles. Fortunately, the safest and most frequently used pathways in neurosurgery via the superior surface of the skull that aligns with the cylindrical MRI scanner bore. Choosing trajectories between sulci minimizes conflicts with veins in a region with otherwise few arteries.

Brain guides for setting catheter trajectories to brain targets are shown in the center column of Figure 2. Imaging MRI‐visible fiducials in the brain guide allows co‐registration of the patient's geometric coordinate system, as defined by MRI, with the surgical environment's coordinate system. The dominant clinical trajectory guide is provided by ClearPoint Neuro [38, 39, 40], as shown in Figure 2D,E. ClearPoint's SmartFrame includes fiducials in its base and surrounding its orientable cannula through which devices are inserted. Co‐registering the cannula is done primarily using 2D cut‐planes through the long axis of the cannula where the water‐filled outer annulus is clearly visible. Through iterative imaging and device manipulation, the line of the desired trajectory becomes aligned with the void in the cannula. Although the Navigus device from Medtronic has a similar MRI‐visible cannula and is FDA‐approved for MRI (Figure 2C), there is no commercial navigation product for it. Thus, its MR usage has been limited to pre‐clinical experiments using customized, real‐time imaging approaches that exploit the symmetry of the cannula to update its position at five frame/s in the field of bio‐psychiatry. The FDA‐approved ClearPoint SmartFlow catheter has been used in numerous clinical studies, elaborated on later in Table 1, and is also used with several pharmaceutical partners at various pre‐clinical and clinical milestones. Typical endpoint catheters are shown in Figure 2F. The 0.7 mm diameter ClearPoint SmartFlow catheter is stepped down to 0.3 mm at the tip to create a structural step that mitigates backflow along the exterior to the catheter. A different approach, where the infusate is pushed through a porous membrane at the distal end of the catheter, has demonstrated larger infusions volumes in canine models [41]. The hole size in the porous membrane (10–30 um), number of holes per cm, and length of the porous region is customizable in the design being developed by Occam (Louisville, KY) [35].

**TABLE 1:** Clinical trials in diseases for which genetic correction is largely required only over a localized region are easier to design.

Disorders	Brain region	Trial	Clinal dose (ml)	Target volume (ml)	Estimated interstitial volume (ml)	Treatment %
Parkinson's disease	Subthalamic nucleus (STN)	NCT00195143	0.05	0.400	0.080	62.5
Parkinson's disease	Putamen	NCT00229736	0.90	5.00	1.00	90.0
AADC deficiency	Substantia nigra	NCT02852213	0.05	0.122	0.0244	200.0
AADC deficiency	VTA	NCT02852213	0.03	0.090	0.0180	167.0
Malignant glioma	Recurrent tumor site	Patel el al. 2016	2.40	2.400	2.400	100.0

*Note*: The clinical infusion volumes (Dose), volume of targeted brain region, and the estimated interstitial volume (20% of the Target volume) are listed for 5 example trials with localized targets. An estimate of the percentage of the target that was likely treated, generated by dividing the clinical dose by the estimated interstitial volume within the target, region as shown in the last column [14].

## 4 MRI Infusion Monitoring

CED catheters are connected to computer‐controlled infusion pumps that modulate hydrostatic pressure to maintain a constant flow rate. Flow rates typically range between 1 and 30 μL/min, with tissue distribution volumes achieving a ratio of 3:1–5:1 relative to the infusion volume [14]. However, distribution can be influenced by factors such as infusate loss along ependymal or pial surfaces, preferential flow within PVS spaces, and variations in isotropic properties at gray/white matter boundaries.

MRI enhances the overall success and reliability of gene therapy procedures by providing continuous monitoring throughout the intervention [32]. Conventional 3D T1‐W methods are adequate for high‐resolution monitoring of the slowly growing infusion region [32], especially when the therapy is co‐infused with non‐ionic Gd contrast agents. In some cases, developers of gene therapies and stem cell therapies for neurodegenerative diseases prefer to avoid co‐infusion with Gd tracers. In these infusion designs, one can track the growth of the infusion zone using T2‐W methods, albeit with less resolution and longer imaging times [42, 43].

However, the proven PVS clearance of the minute levels of Gd co‐infused in CED trials, the safety of non‐ionic contrast agents [44], and the desire to accelerate surgery have repeatedly led to FDA approval of Gd tracers in adult and pediatric trials using CED (NCT02022644, 01156584, 03065192, 03562494, 02852213). The amount of co‐infused Gd needed in a large gene therapy treatment is still only on the order of 1/1000 of the Gd dosage used in a single intravenous infusion. Numerous pre‐clinical studies have demonstrated the excellent concordance of the infusion distribution volume, as demonstrated by T1‐enhancement with a Gd contrast co‐infusion, with gene expression as demonstrated by histopathology [26, 44, 45] or small molecule distribution in CED cancer‐based therapies [46, 47].

There is also growing concern about gadolinium deposition in the brain though the long‐term implications of this are not yet fully understood [48]. The accumulation of gadolinium in these studies, however, is attributed to numerous intravenous injections of MR contrast over years [49] where some small percentage eventually finds its way to the brain through pathways not fully understood. In CED trials, the Gd tracking effort is only adding 1/1000 of a typical intravenous Gd dosage, and serial imaging after the infusion demonstrates full clearance [50]. Alternatively, non‐contrast methods to visualize infusions can be achieved via specialized scan parameters [45, 46] for cases wherein the trial designers are still concerned about the potential effects of Gd.

Real‐time MRI has enabled neurosurgeons to refine their techniques during early trial phases to improve target coverage minimizing off‐target distribution, backflow along the cannula tract, and loss of infusate into non‐target regions like ependymal or PVS spaces. Strategies to cover the ellipsoidal putamen in PD trials are a specific example [38]. Additionally, intraoperative imaging allows for standardized comparisons of procedural effectiveness, including target coverage rates, across surgical centers and clinical trial participants.

## 5 Trials Using Localized Gray Matter Delivery

CED infusions initiated in gray matter expand approximately spherically until they reach white matter. If the targeted volume is contained to 1 gray matter region, executing a CED infusion is much simpler than a broader infusion. As the interstitial pore fraction of gray matter is approximated at 20%, infusion volumes of 1/5 of the targeted treatment zone volume would, theoretically, be sufficient. In practice, clinical trials use larger infusion volumes to ensure complete coverage.

Examples of clinical trials attempting to treat relatively small gray matter regions, shown in Table 1, list the targeted infusion region, clinical infusion dose volume, the volume of the targeted region, the estimated volume of interstitial pathways within the target region assuming a 20% pore fraction, and the ratio of the estimated interstitial volume to the infused treatment volume, which we term the Treatment % in Table 1. At the smaller end, successful AADC deficiency trials required infusion volumes of only 0.05–0.10 mL [14] to cover the substantia nigra, the regions targeted to enable a child to generate dopamine. Applications targeting the subthalamic nucleus for Parkinson's Disease required a similarly small infusion. Larger, yet still manageable, infusion volumes of 0.45 mL per hemisphere have been used in PD trials targeting the putamen. Current MRI imaging technology, catheter technology, and neurosurgical techniques are adequate to cover localized gray matter regions minimizing problematic off‐target delivery.

The last column of Table 1 shows significant variation in the design of infusion protocols to treat each brain region [14]. The entries for Parkinson's Disease in Table 1 indicate that the infused dose was not large enough to cover the entire putamen. Larger doses are often needed for full target anatomical coverage due to backflow and irregular target distribution shapes. For example, clinical trials targeting the putamen showed only about 50% [14] of the target volume was covered due to losses from backflow. In some gene therapies, the scientific design compensates for this by planning a larger margin for the treatment zone. In cancer convection‐enhanced delivery (CED), larger dosages are usually used to completely cover targeted brain volumes.

## 6 Difficulty of Treating White and Gray Matter Simultaneously

**FIGURE 3:**
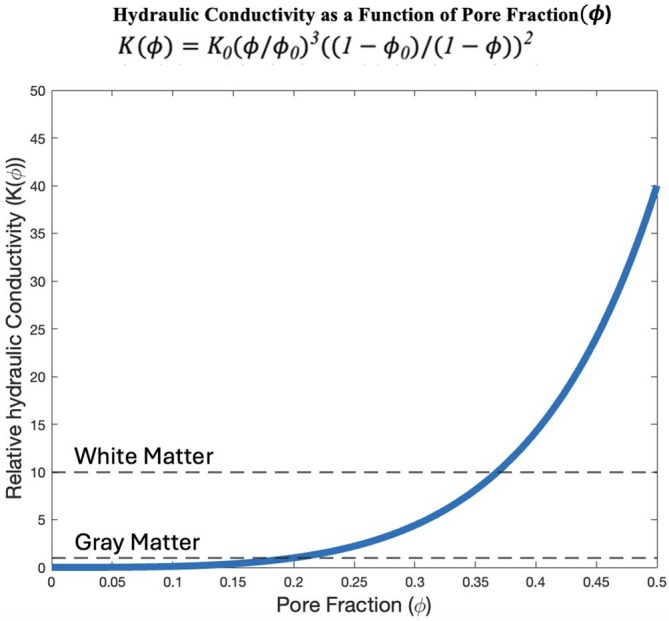
The pore fraction in gray matter and white matter can be approximated at 20% before the pressure of the convection‐enhanced delivery (CED) infusion begins [51]. Although the pore fraction varies little in gray matter during CED, the more organized white matter with its myelinated tracts can realistically double its pore fraction during CED. If the hydraulic conductivity of gray matter is normalized to 1, this illustration shows the relative advection rate of the infusion in white matter can easily be 10 times faster than gray matter [52, 53]. This has led to the impression in the pharmaceutical world that CED infusions starting in gray matter expand until a white matter tract is reached, upon which the rest of the infusion is constrained to white matter. The blue line represents the mapping from pore fraction to hydraulic conductivity.

Under the interstitial pressure created during CED, the infusion will go where tissue resistance is lower. Although the interstitial space in gray matter is relatively unorganized, the myelination in white matter creates an organization that allows the interstitial space to relax and expand under pressure much more easily than in gray matter. Figure 3 demonstrates that the relative speed of growth of the infusion front in white matter can be 10 times faster than in gray matter. Once an infusion that starts in gray matter finds a white matter tract, the infusion will preferentially shunt into the white matter tract. There is no way for a neurosurgeon to visualize the likely pressure front during the infusion and thus estimate the final infusion distribution simply by looking at MR structural images.

**FIGURE 4:**
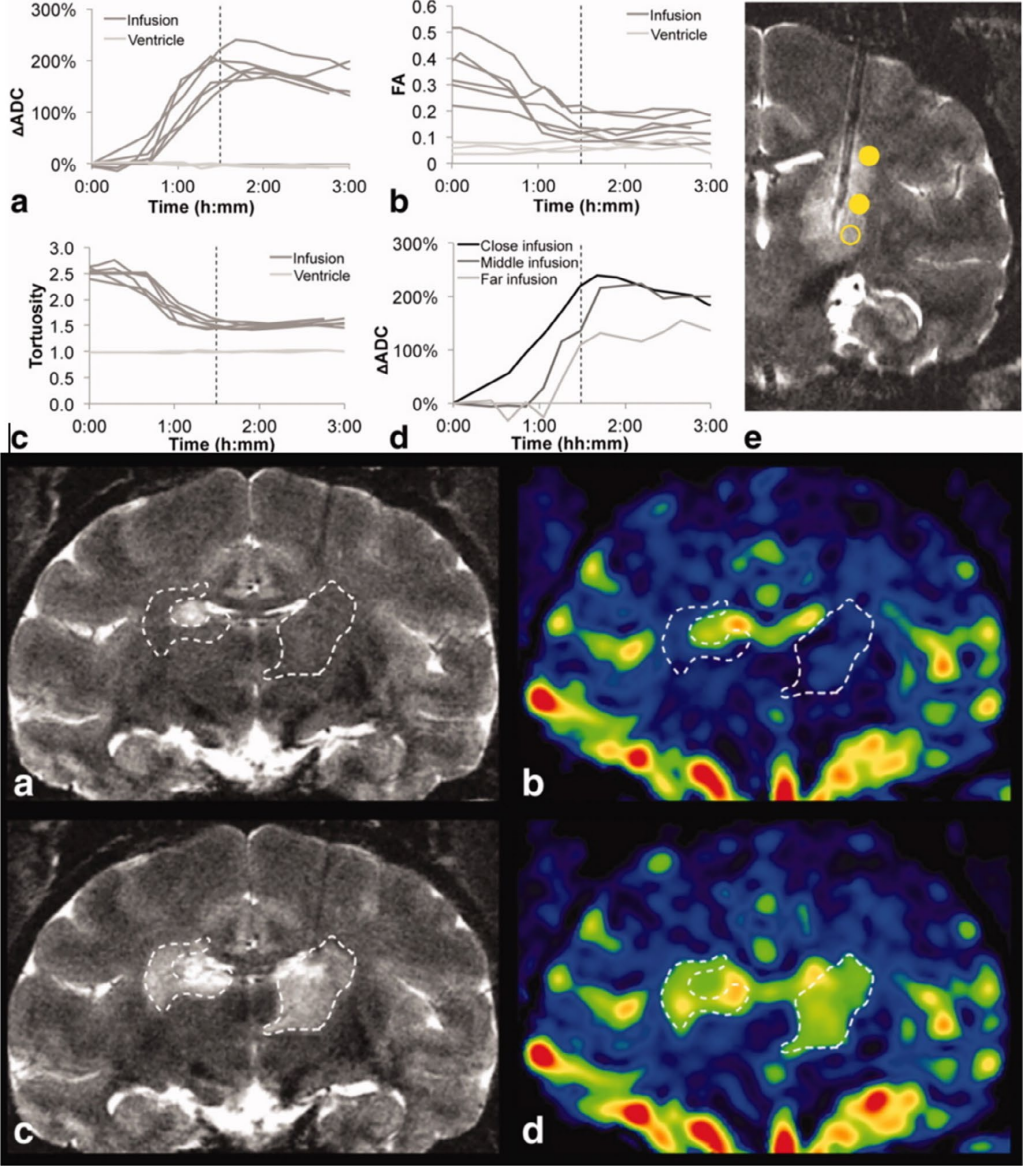
(A) Demonstrates apparent diffusion coefficient (ADC) increase (B) The fractional anisotropy (FA) decreases with respect to time, demonstrating the fluid build up in the brain. (C) Describes the decrease in tortuosity of white matter tractks involved in the winding paths of the infusion therapy in the brain. (D) Demonstrates close infusion expanding with time for the apparent diffusion coefficient (ADC).

The signal to noise ratio of 7T MRI was exploited to track the progression of CED brain infusions in real time in non‐human primate (NHP) models (Figure 4). High‐resolution T2‐W and diffusion‐tensor images were used to show decreases in fractional anisotropy and increases in the apparent diffusion coefficient (ADC) in white matter during the infusion. Such methods are one way to model how advection rates in white matter change as the region of high pressure increases as the infusion grows [42].

**FIGURE 5:**
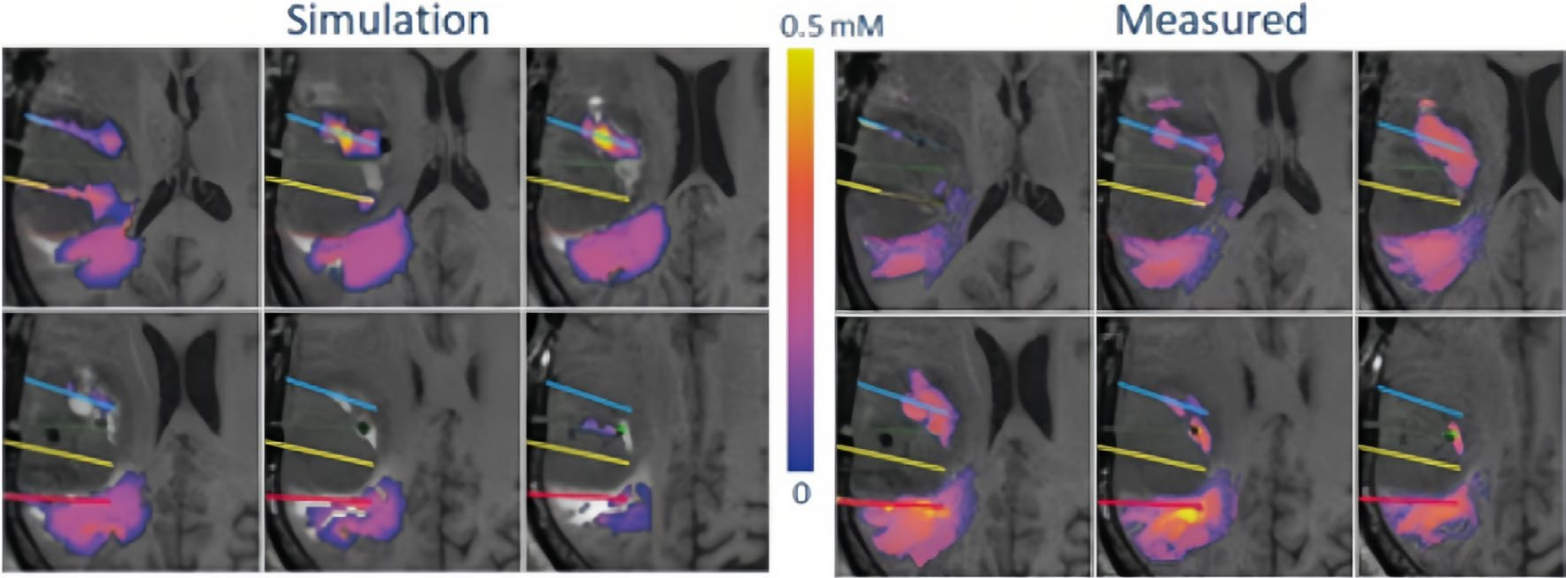
Six axial slices demonstrate the infusate front of convection enhanced delivery infusion (color overlay) at the 54‐h mark of a 72‐h infusion in an adult brain cancer patient. Four catheters (blue, green, yellow, and red projections) each infuse at 10 μL/min. Remaining differences, particularly in the posterior portion of the fifth and sixth slices, show the importance of the proposed real‐time monitoring. Figure adopted with permission from Therapeutic Delivery [55].

Sophisticated neurosurgical trials in treating recurrent brain tumors have addressed the preference of CED for advancing along white matter tracts by using an FDA‐approved tool to forward model the likely end infusion coverage for a set of given catheter locations, flow rates, and infusion duration [54, 55]. iPlanFlow [52], a BrainLAB offering, is built off the algorithm created by Raghavan and Brady based on the biophysics of flow through a porous medium, the brain in this case (Figure 5) [23]. Significant improvements to the methodology were provided by Therataxis, Raghavan, and Brady with its Molecular Flow Simulation (MFS) tool, also FDA‐approved. The predicted treatment region, shown as a color overlay in Figure 2A, often closely matches the final infusion zone, shown as the contrast‐enhanced region in Figure 2H. In past brain cancer trials, neurosurgeons iterated on a set of catheter locations until the pre‐surgical prediction of the final infusion region matched the desired treatment zone about the recurrent tumor.

The current generation of CED simulators is completely dependent on MRI to provide surrogates of the interstitial flow parameter maps that generate the stepwise, anisotropic growth of the infusion front during a CED infusion.

Although brain cancer trials have exploited these tools to treat the heterogeneous territories around brain tumors, segments of the pharmaceutical industry working on whole brain rare neurodegenerative diseases are unfortunately at a much earlier stage and must concede that treating white and gray matter simultaneously is difficult with CED. Recent clinical trials have aimed to only treat white matter, even though the disease damage is often centered in gray matter [14].

## 7 Rare Genetic Disease Gene Therapy Trials Needing Broad Coverage

**FIGURE 6:**
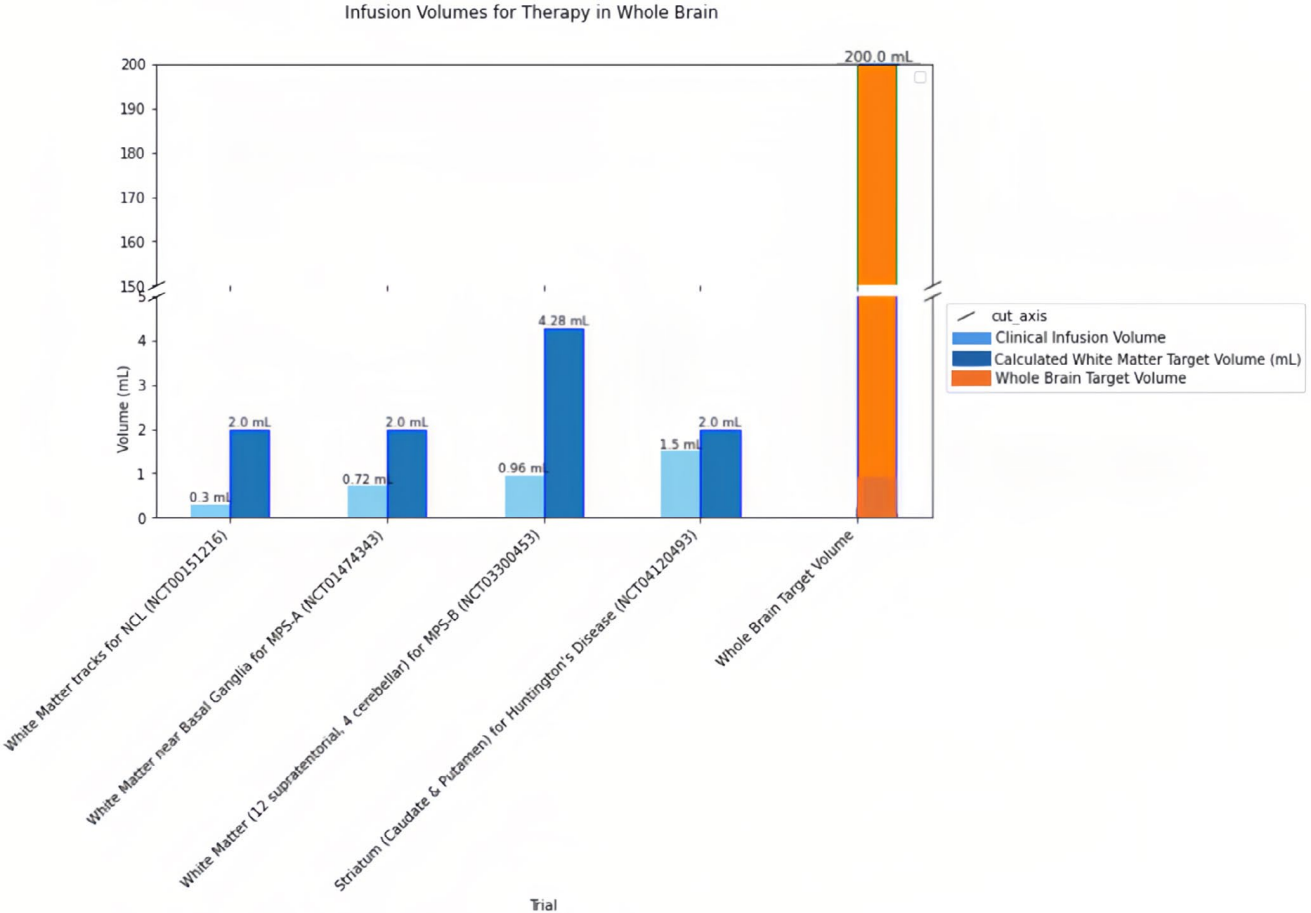
Infusion volumes and total treated volumes of specific brain regions for clinical trials of rare disorders that affect the whole brain: Batten (NCL), MPS‐A, MPS‐B, and Huntington's disease. The desired improvement in clinical outcomes in these trails is modest due to the significant differences between the treated white matter (dark blue bars) and the larger volume of the entire brain (orange bar), whereas the actually clinical infusion volumes used (light blue bars) don't completely cover desired target volumes (dark blue bars).

Gene therapy strategies for monogenic lysosomal storage diseases like variants of Batten Disease (Neuronal Ceroid Lipofuscinosis or NCL) and San Filippo (mucopolysaccharidosis MPS III) are often straightforward and successful locally where administered. Figure 6 shows an example of recent trials to administer gene therapies using CED in Batten, San Filippo, and Huntington's Disease. As shown in Figure 6, the volume of the treated white matter tractks regions is an order of magnitude smaller compared to the entire brain volume. The incremental design of trials is partially due to a lack of collaboration between the gene therapy, neurosurgical, CED modeling, and MRI communities.

When broad brain coverage is needed, the clinical trial outcomes are designed only to slow disease progression and not wholly stop the brain damage caused by these genetic errors. The brunt of the remainder of this review explains how MRI is used today to provide the spatial maps that enable the prediction of CED infusion distributions. We conclude with how advanced brain mapping techniques being pioneered by the MR community could be retooled to create a new generation of interstitial flow simulators. These simulators, coupled with more catheters and longer infusion periods, would enable neurosurgeons to plan trials that would treat much broader regions of the brain. The broad MRI community focusing on tracking pediatric brain development from infancy to adolescence could also provide the necessary brain characterization to enable CED at much younger ages before brain damage accumulates.

## 8 Predictive Modeling in Large Coverage Infusion

When the desired infusion covers both white and gray matter regions, it is not possible for a neurosurgeon to predict the end‐distribution simply from their own cognitive model of the brain. As in other areas we have examined here, the failure of some of the first CED brain cancer trials was attributed to poor drug distribution as shown in Duke's PRECISE trial [17]. Pre‐surgical forward CED modeling later proved helpful in allowing surgeons to test out catheter tip locations, flow rates, and infusion durations and visualize the likely final biologic distribution volume in brain cancer.

As shown earlier in Figure 3, the advection rate of CED infusions will increase dramatically in white matter staying largely unchanged in gray matter. Infusions that reach cerebrospinal fluid (CSF) regions such as sulci and large PVS spaces, modeled as a region of zero pressure, are shunted into the CSF. MRI, as shown in Figure 5, provides spatial maps that enable CED prediction algorithms to model flow through the poroelastic brain [52]. T1‐W, T2‐W, and DTI are used to segment the brain into white matter, gray matter, and CSF compartments. In the case of brain tumors, dynamic contrast‐enhanced (DCE) MRI demonstrates regions where the BBB has been compromised and thus infusions will shunt [56, 57]. This added mapping is generally not needed when the BBB has not been compromised.

**FIGURE 7:**
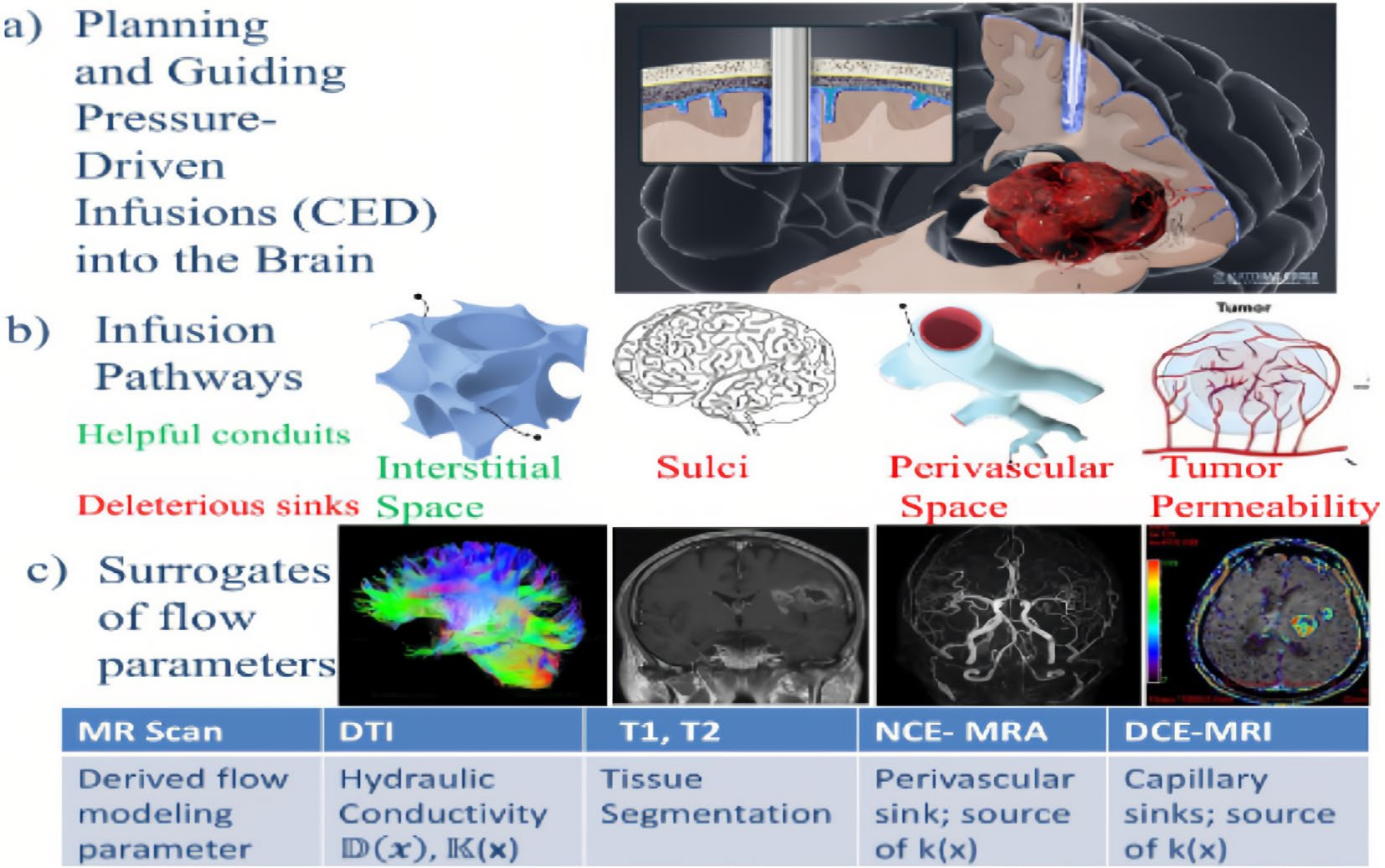
Illustration of the required parameters gathered from different MR acquisitions to predict and plan guided, pressure driven infusions with convection‐enhanced delivery (CED) into a brain tumor. Such parameters include infusion pathways obtained from structural MR imaging and surrogates for pre‐monitoring infusate predictive modeling.

As shown in Figure 7, MRI is used to create spatial maps that translate MR diffusion parameters into a hydraulic conductivity tensor map, denoted by the tensor **K** in Figure 7 [55, 58].

**FIGURE 8:**
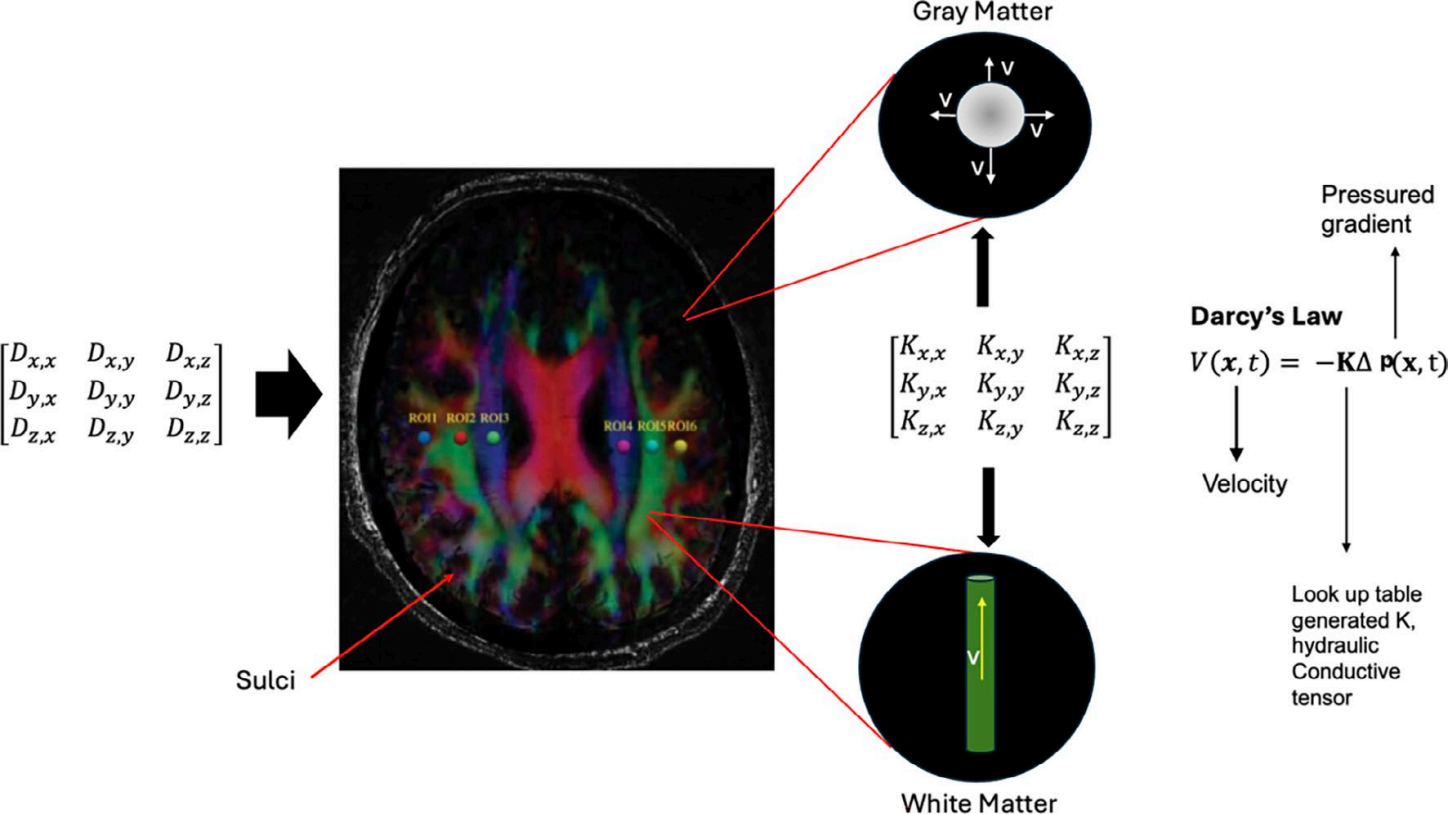
Lookup tables are used to convert inputs from MRI diffusion tensors into the hydraulic conductivity tensors needed to calculate the spatially varying velocity of the growing infusion front. Iterative optimizations use Darcy's Law to update the velocity profile of the infusate based on the initial pressure gradient at the tip of the catheters and the hydraulic conductivity tensor from the diffusion tensor. Although the directional information of **K** consistently aligns with the MR diffusion tensor, the algorithm increases the magnitude of **K** elements as pressure increases in each spatial region, increasing the pore fraction, as shown earlier in Figure 3.

As illustrated in Figure 8, the fundamental form of Darcy's Law governs flow in a porous media. This is the fundamental law used to predict the infusion's 3D velocity profile during CED. Here, *V* is the Darcy velocity (or specific discharge), **K** represents the extracellular hydraulic conductivity and ∆p denotes the pressure gradient driving the flow. The negative sign reflects the flow direction being opposite to the pressure gradient (Figure 8). The anisotropic direction of infusion expansion will not change within a white matter tract, but the magnitude of the velocity of the front will grow with pressure, as shown earlier in Figure 3. The prediction algorithms thus update the **K** tensor map at each time point.

The iterative algorithms need an initial estimate of the pressure distribution at the catheter tip to make a first estimate of the varying velocity at the outside surface of the growing infusion. using Darcy's equation is used to relate velocity to pressure. Knowledge of the actual current infusion volume at each time step is used to refine the pressure and velocity estimates to create a realistic solution. This pressure approximation is prone to error and depends highly on the catheter tip geometry. Unfortunately, the iPlanFlow software has not been updated to be compatible with many of the catheter tips used in clinical trials today. Instead, real‐time MRI monitoring can create a 3D map of the velocity of infusion growth to produce a better spatial approximation of pressure about the catheter tip. Figure 8 shows how simply subtracting the region of T1 enhancement, created by the previously mentioned co‐infusion of a Gd tracer, at two early time points within the first 30 min of the infusion protocol provides the needed 3D map of initial infusion velocity [59, 60].

**FIGURE 9:**
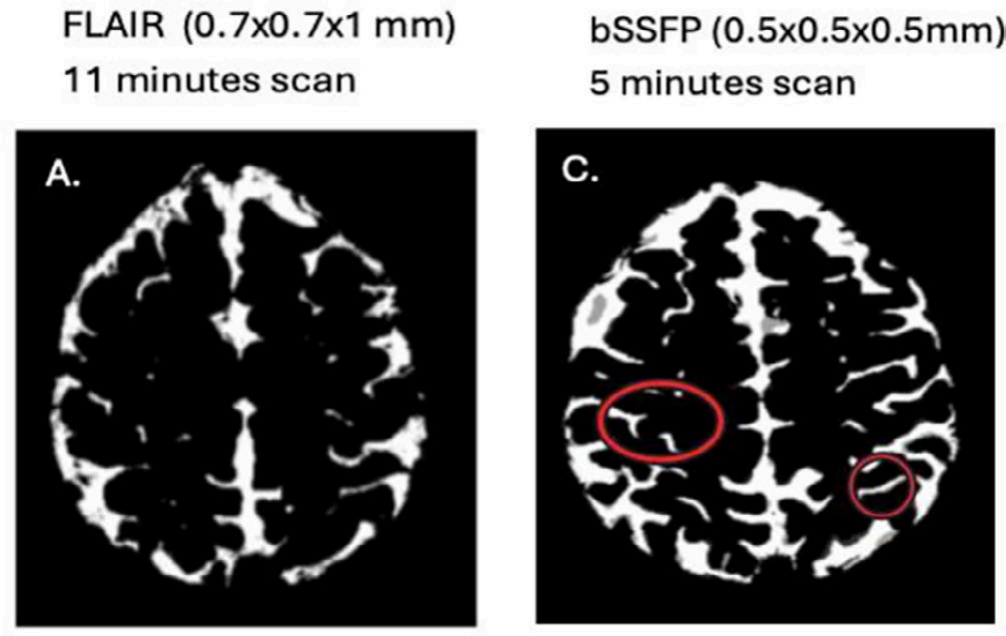
Illustration shows better delineation of sulci maps in 5‐min positive contrast bSSFP scans over a standard negative contrast 11‐min scan from FLAIR [63].

The rapid growth of gene therapy treatments in the brain will permit customized presurgical MRI exams instead of the standard diagnostic MRI protocols that generate conventional T1, T2, and DTI volumes today. For example, a family of 3D structural RARE‐based inversion recovery sequences [61] that produce high resolution 3D gray matter only volumes has been gaining utility in the placement of neuromodulation devices. These would be advantageous for gray matter segmentation for predictive CED infusion algorithms. Likewise, infusion prediction algorithms would benefit from higher resolution methods of detecting sulci, the low‐resistance pathways that shunt infusions. Current commercial algorithms rely on conventional, negative contrast imaging (T1‐W FLAIR, Turbo FLAIR, FLAIR TSE) that need significant morphological processing to derive the necessary sulci maps. Conversely, balanced steady‐state free precession (bSSFP) provides [62] high positive contrast between cerebrospinal fluid (CSF) and adjacent gray and white matter, allowing for a more precise and easy separation of sulci via rudimentary thresholding. This approach effectively simplifies the required image processing also lessening the amplification of noise from morphological edge detection. An example comparing the standard T1‐W methods with bSSFP showing enhanced sulci detection is shown in Figure 9.

## 9 Next Generation Characterization of Interstitial Spaces for CED

**FIGURE 10:**
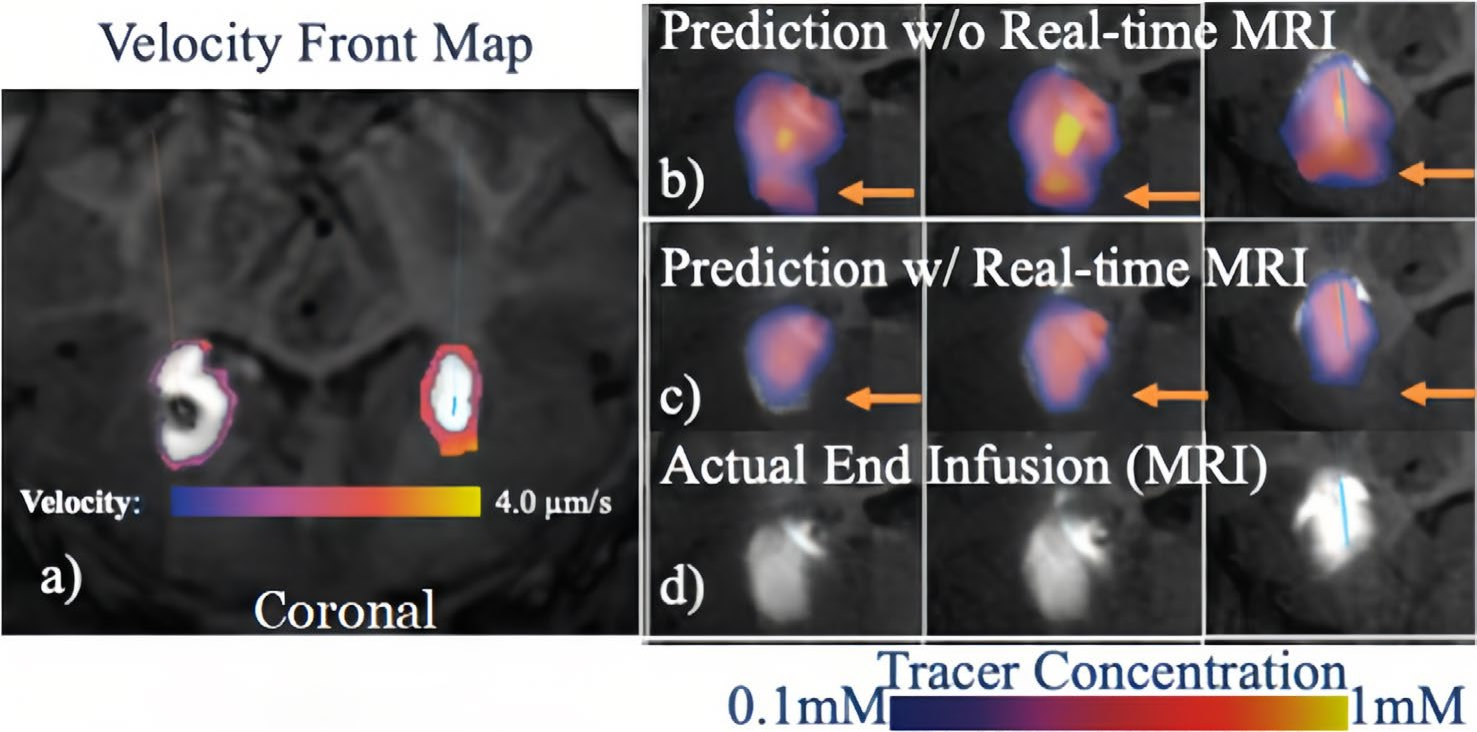
Illustrations in a swine model shows how the pre‐surgical color overlay of the final infusion prediction (b) overestimates the total infusion volume at the inferior end of the infusion, when compared to the actual end infusion shown in Row (d) A better prediction, especially in area of the orange arrows, is overlaid over the final infusion zone in Row (c). The Row (c) overlay closely matches the actual grayscale infusion, again as shown in Row (d). The improved prediction is achieved by replacing assumptions on the initial pressure about the endport catheter tip with a real‐time 3D estimate of the velocity of the infusion front early in the actual surgery (Row A). The Velocity Front Map is generated by subtracting the infusion images at 10‐min intervals during the first 30 min of the infusion. The color map in (a) shows different velocities spatially about the catheter tip early in the experiment.

In CED within white matter, the infusate advances along the axons in bulk through the extra‐axonal compartment, as the organization of myelin allows the infusate to expand more easily under pressure, therefore making flow in white matter preferred. The armentarium of methods developed for sensitizing MRI to myelin has made MRI the dominant, non‐invasive modality for monitoring brain development. Numerous studies have mapped the trajectory of myelin development using multi‐shell diffusion MRI, revealing changes in white matter microstructure that influence infant functional development within the visual pathway [64]. DTI tractography and 3D spoiled gradient recalled (SPGR) quantitative susceptibility mapping (QSM) studies have shown the potential to characterize white matter changes associated with behavioral improvements in children with Cerebral Palsy [65]. MRI studies evaluating deep gray matter regions showed significant differences in volume and mean kurtosis between healthy controls and patients with amnestic mild impairment (aMCI)/Alzheimer's disease. New diffusion MRI techniques, such as diffusion kurtosis imaging (DKI), bi‐tensor DTI, and neurite orientation density and dispersion imaging (NODDI) have provided a valuable addition to conventional DTI for the evaluation of neurodegenerative diseases [66]. Algorithms such as iPlanFlow and the Molecular Flow Simulator treat all white matter as equivalent in their initial computation before manually reworking CED predictions in regions of high neuronal density like the corpus callosum (see Figure 10, green).

Separating the extraaxonal compartment from other cellular structures is a task not generally needed in tractography [67]. Proper characterization of the extracellular compartment would provide a much more direct measure of poroelastic parameters than the lookup tables that map conventional DTI to hydraulic parameters in today's predictive CED models.

As conventional diagnostic MRI has not mapped extraaxonal water separately, CED infusion parameters have initialized the interstitial pore fraction at a spatially invariant level of 20%, a consensus figure derived from invasive physiologic techniques [9]. The elasticity index shown earlier in Figure 3 is adjusted iteratively within the prediction algorithm to make the best estimate of the final infusion distribution that matches other infusion constraints [68].

Fortunately, advanced MRI gradient systems and DTI modeling methods are fitting more and more parameters to characterize the human brain. Generally, these methods have fit additional parameters to improve white matter tractography, but the additional parameters could be utilized to provide more direct characterization of hydraulic conductivity in the brain.

For example, the quantitative MR neurite characterization model known as NODDI has achieved significant attention in neuroscience research [62, 69, 70]. As NODDI models were designed with tractography in mind, intra‐axonal signal contributions were typically lumped in with extracellular contributions.

**FIGURE 11:**
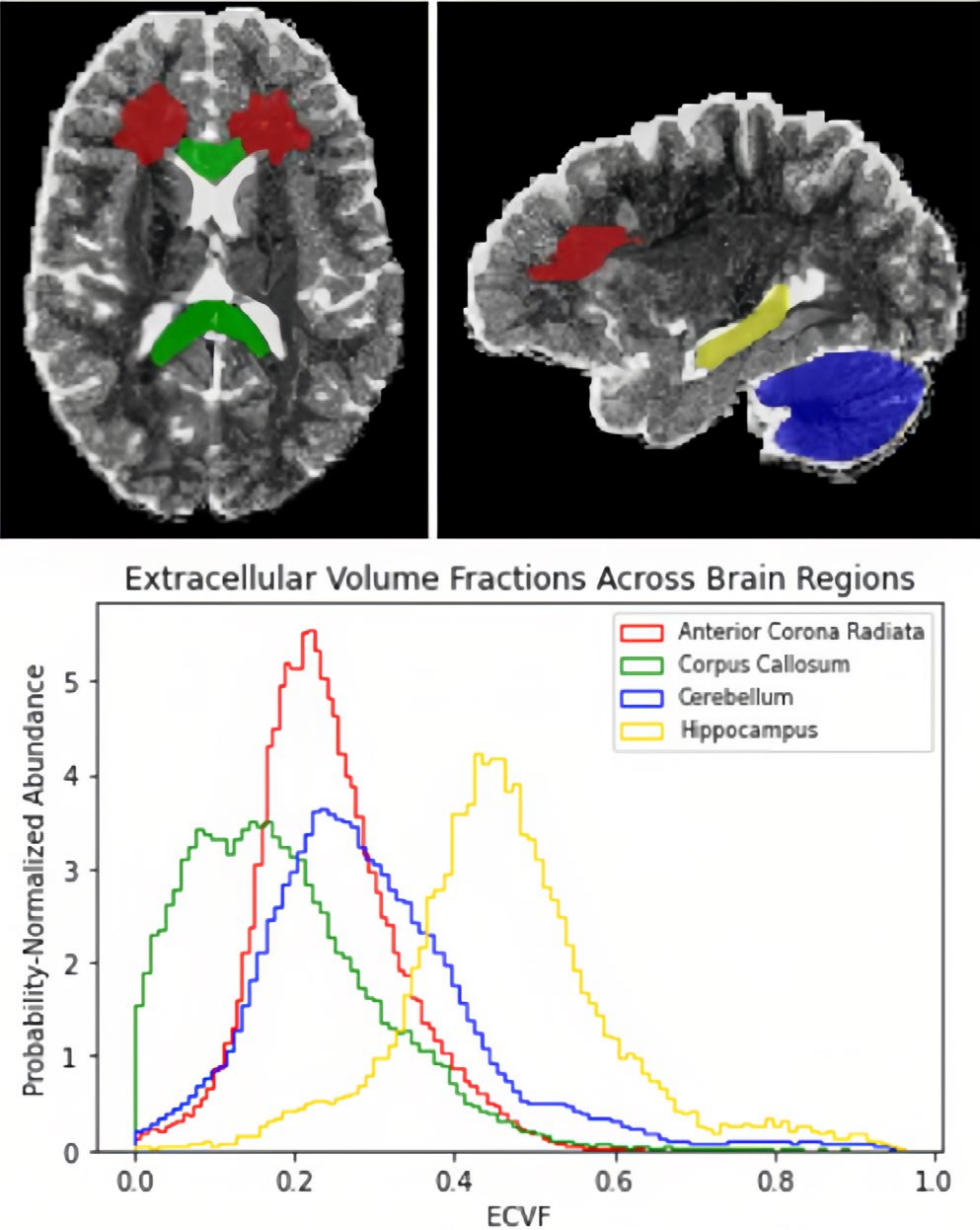
Current infusion prediction algorithms are initiated with a spatially‐uniform estimate of interstitial pore fraction. Measures of the extraaxonal compartment are likely a more relevant initial parameter, as this is the space in which the infusate buffer can travel along white matter. Here, histograms of the extracellular volume fraction (ECVF), as provided by the soma and neurite density imaging (SANDI) model, within contours of the anterior corona radiata (ACR), corpus callosum (CC), hippocampus (Hp), and cerebellum (Cb). Contrary to the spatially invariant assumption used today, there is considerable spatial variations in the ECVF compartment [71].

More recent diffusion models potentially offer more direct utility for mapping the hydraulic parameters needed in predictive modeling of CED distributions [55]. Soma and Neurite Density Imaging (SANDI) adds a compartment to the diffusion model to separate the signal from neuronal somas (cell bodies) and neurites from extraaxonal water, allowing greater specificity to evaluate microstructural environments, as shown in Figure 11 [68, 72]. The lower level of extraaxonal water in the corpus callosum in Figure 11 (green curve) is consistent with the pore fractions stated in Figure 3. This aligns with the experience of neuroradiologists, who understand that edema from brain cancer will shunt away from the corpus callosum.

## 10 Exploiting MR Field of Glymphatic Flow for Acceleration CED Distribution

Changes in CSF motion due to cardiac pulsatility, breathing, [72] and slow wave sleep have been posited as hypotheses by which peristaltic motion in arterial and PVS spaces could alter the clearance of brain waste. In CED, MR's ability to image neurofluids could be used to develop infusion protocols to enlarge distribution volumes. For example, cardiac pulsatility is already known to improve the distribution of gene therapies in studies comparing CED infusions in live and recently euthanized animal pre‐clinical models [73]. Recent evidence in animal models of the theory of how neurovascular coupling modulates CSF flow creates a new potential application for advances in the sensibilization of MRI to neuromodulation [74].

The MRI community is already playing a seminal role in developing imaging methods sensitive to the slow motion of CSF in the brain and its exchange with the interstitial spaces of the brain [75, 76, 77]. Recently, the concepts of T2 preparation and CSF nulling were advanced to study CSF/brain exchange in whole brain studies. MRI's ability to simultaneously image incoherent and coherent motion in the brain [78] resolving cardiac and respiratory periodicity could prove instrumental in designing infusion protocol enhancements.

## 11 Recommendations for MRI Community to Advance Neurodegenerative Gene Therapy

A significant segment of the MR community is working on platform technologies to advance neurological imaging, diffusion imaging, general myelin characterization, [70, 79, 80] and high‐resolution anatomical imaging. The community is encouraged to look for new opportunities for its advances that can be used to improve the understanding of how CED infusions can better predict final infusions and treat wider distributions. For example, we described how the often‐overlooked compartment of extraaxonal water is particularly important in CED. MR mapping of this parameter would provide better assumptions of initial conditions, improving the early iterations of the predictive modeling, enabling faster model convergence, and removing the need for manual correction in areas of higher neuronal density.

The primary focus of MRI DTI has been white matter for obvious reasons. However, curing many of the rare neurodegenerative genetic diseases will require a better understanding of the interstitial spaces in gray matter [81]. The application of high‐end diffusion imaging systems may provide the ability to better characterize the smaller cellular dimensions in gray matter.

Gene therapy in the brain for rare pediatric neurodegenerative diseases could leverage lessons learned from the rapid advance of spinal cord treatments for SMA. Here, treatment protocols aim for the first few months of life the spinal cord is smaller, less myelinated, and undamaged. Similarly, outcomes for whole brain diseases like fatal neurodegenerative lysosomal storage diseases will improve if carried out before damage accumulates in smaller, less myelinated brains. The infant brain grows from 350 to 400 g at birth to 80% of its adult weight by year two [80], as shown in Figure 11. Myelination of white matter also rapidly develops over this period, as further shown by the FA maps of Figure 11. Clinical trials like those in Figure 6 would obtain much greater coverage if performed soon after birth on smaller brains. Designing trials with broader coverage would be easier if the reduction of myelin reduced the difference in advection speeds between gray and white matter.

Although the genetic screening of many rare diseases is not widely performed at birth today, the practice is growing. The experience with SMA indicates that as treatments become available, the pressure for governments to screen at birth to permit earlier treatment grows dramatically.

**FIGURE 12:**
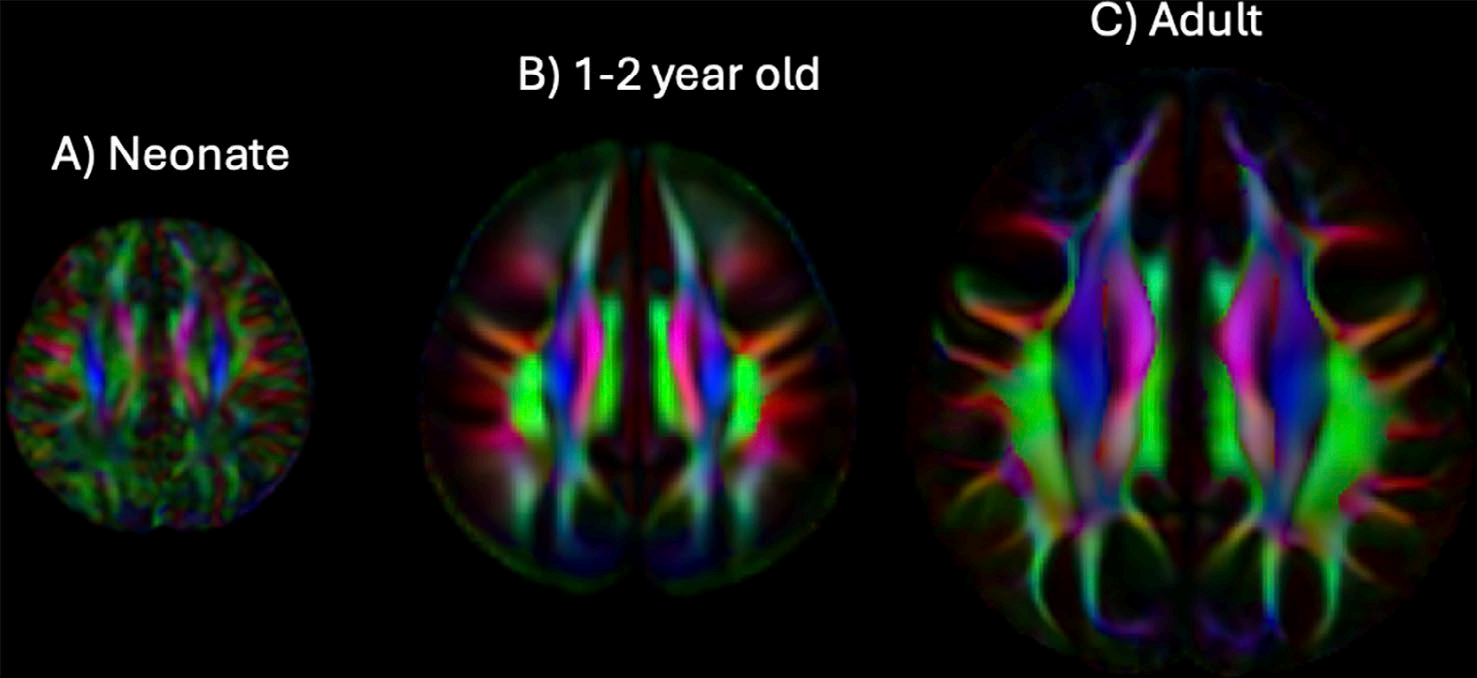
Comparison of fractional anisotropy (FA) maps shows rapid overall growth and myelin development from a scanned (A) neonate, (B) 1–2‐year‐old children to (C) adult [79, 86, 87].

CED infusion prediction algorithms would need to be updated with segmentation methods and hydraulic conductivity parameters tuned to the infant brain. Fortunately, numerous studies have characterized myelin development [70, 80] early on in child development. Larger multisite trials are obtaining longitudinal, multi‐shell diffusion data from birth through adolescence [76, 82, 83, 84, 85] (Figure 12). Magnetic resonance imaging (MRI), particularly advanced diffusion‐weighted imaging (DWI) and DTI, plays a critical role in characterizing these parameters. The data in these studies could be analyzed to provide the interstitial flow parameters to enable accurate prediction algorithms at the earliest stages of child development. Simulated CED prediction algorithms could predict likely brain coverage for sets of catheter trajectories deemed safe and feasible by pediatric neurosurgeons. If generated early on in a pharmaceutical development cycle, the simulations would give much better indications of likely clinical outcomes than are possible today.

The cerebellum is often an overlooked application of MRI, but its study is crucial to devise strategies for treating numerous rare ataxias [88]. The inherently higher heterogeneity of interfaces of white matter, gray matter, and CSF and the smaller spatial scale of features make the cerebellum a prime application for high‐field magnets and premium gradient systems used for high‐performance DTI.

There is significant demand from pharmaceutical companies for surrogate biomarkers of therapeutic efficacy and matching investment from public governmental bodies. The FDA Critical Path program has created two consortiums, one in rare diseases and another in lysosomal storage diseases, which concentrate efforts in advancing neurological biomarkers.

Although the presence of molecular markers of genetic repair in CSF and blood samples provides some evidence of efficacy, MRI is still the dominant way of spatially mapping the effects of gene therapy on advancing normal brain development and/or slowing degeneration. At the simplest level, real‐time MRI of gene therapy infusions will provide potent evidence of treated regions. Methods to correlate these regions with normalized brain function and/or development will be highly prized in the pharmaceutical field and with patient advocacy groups.

## 12 Conclusion

MRI‐guided intraparenchymal gene delivery has already played a key role in guiding and monitoring key clinical trials that are moving treatments for neurodegenerative diseases from palliative care to cures. Families previously with little hope for their children are now anxiously tracking new trials.

Catheter guidance technology and MRI infusion monitoring methods are generally adequate when the treatment aims to treat local regions situated in gray matter. MRI plays a key role in providing mappings that allow prediction of therapeutic coverage when genetic correction over wider regions, encompassing gray and white matter, is needed. Here, further advances in the application of MRI to guiding intraparenchymal delivery are needed. Advanced neurological MRI methods for segmenting the brain and characterizing its hydraulic parameters, particularly at very young ages when myelination development is initiating, are needed. Fortunately, many existing efforts in advanced diffusion imaging and longitudinal tracking of child development can likely be leveraged.

## Conflicts of Interest

Walter F. Block and Tom Lilieholm has ownership and leadership positions in IMGGYD LLC. The aims of the company are to develop drill guides and other devices for supporting MRI‐guided surgery. Walter F. Block is also a paid consultant for Turing Medical and Co‐Investigator on a NIH SBIR funded grant from Turing Medical. The other authors declare no conflicts of interest.

## Supporting information


Data S1.
