# Supplementary material for: MR‐Guidance of Gene Therapy for Brain Diseases: Moving From Palliative Treatment to Cures
Source: J Magn Reson Imaging. 2025 Apr 21;62(5):1280–95. doi: 10.1002/jmri.29804 (PMC12354169; doi:10.1002/jmri.29804)
Supplement: Supplementary file 1 — Data S1. [file JMRI-62-1280-s001.docx]

Dear Reviewers,

Thank you for the comments on the paper as we fully considered each one of them to improve our paper. We were highly encouraging by Reviewers 5 comment on the manuscript being “forward-looking and meaningful achievement”. We have in green underscore all grammatical chances, highlighted in yellow all new added information’s, and highlighted in blue all new information pertaining to MRI related information. Substantial fixes of grammar make it seem like whole document content has been completely rewritten. However, this is not the case. Only MRI content was added as suggested and fixed specific review critiques. In addition, we have added 19 references from jMRI and 9 references from MRM.
